# Changes in appetite-related hormones may not necessarily lead to changes in appetite response

**DOI:** 10.1590/1806-9282.20250410

**Published:** 2025-09-19

**Authors:** Weijia Meng, Zheying Jiang, Cuiping Li, Jingjin Yang, Huaqin Su

**Affiliations:** 1 Taizhou University, Xiachen Community Hospital Affiliated to the School of Medicine – Taizhou, China.

Dear Editor,

We are pleased with the study by Alyar et al.^1^, which showed that the energy deficit created by long-term calorie restriction and exercise increased ghrelin and peptide tyrosine tyrosine levels in individuals with diabetes. However, I think there are still some issues that need to be addressed.

The main problem with this study is that changes in appetite-related hormones do not necessarily cause an appetite response. The study by Alyar et al.^1^ was designed to determine the effect of the energy deficit caused by long-term (12-week) calorie restriction and exercise programs on appetite responses in obese individuals with prediabetes and type 2 diabetes. It is not appropriate to detect appetite-related hormones instead of the appetite response.

In fact, changes in appetite-related hormones do not necessarily cause an appetite response. The study conducted by Nguo et al.^2^ revealed that alterations in appetite-related hormones did not correspond to subsequent changes in subjective appetite or energy intake. Consequently, it is inadequate to assess appetite response solely by measuring variations in appetite-related hormones. Appetite may also be affected by the type of food, personal emotions, and environment^3^. When levels of appetite-related hormones increase, individuals do not demonstrate a pronounced appetite response if the food presented does not align with their preferences. Similarly, even if the levels of appetite-related hormones in the body are elevated, a poor emotional state in an individual may inhibit an increase in appetite response^4,5^. Furthermore, the environment in which an individual is situated can significantly influence the modulation of appetite responses, independent of hormonal changes^6^. For instance, in a noisy and chaotic environment, individuals may become distracted from their physiological hunger cues, even when appetite-related hormones indicate a need for food intake. Conversely, a calm and inviting dining atmosphere can enhance appetite, regardless of current hormonal levels^7^.

Measuring appetite response is a critical aspect of nutritional research and can be approached through various methodologies^8^. These methods can be broadly categorized into subjective self-report measures and objective physiological measures^9^. The author can use visual analog scales (VAS)^10^ to detect the appetite response in obese individuals with prediabetes and type 2 diabetes. VAS can assess hunger, abundance, appetite, and expected consumption and is often used in appetite research^11^.

The study subjects of this paper are obese individuals with prediabetes and diabetes. However, it is important to note that obese individuals frequently experience leptin resistance, which is characterized by elevated levels of leptin coupled with insensitivity to its effects^12^. Given that this study did not evaluate the presence of leptin resistance among the subjects, the observed reductions in ghrelin, glucagon-like peptide-1, and peptide tyrosine tyrosine levels may not necessarily lead to a decreased appetite response. Consequently, fluctuations in appetite-related hormones, including ghrelin, glucagon-like peptide-1, and peptide tyrosine tyrosine, may not accurately represent changes in an individual's appetite response.

In summary, the findings presented by the author are inspiring. However, there are areas for improvement. The study suggests that fluctuations in appetite-related hormones, including ghrelin, glucagon-like peptide-1, and peptide tyrosine tyrosine, do not entirely account for appetite responses. Therefore, it is advisable to incorporate subjective scales to achieve a more thorough analysis.

## Data Availability

The datasets generated and/or analyzed during the current study are available from the corresponding author upon reasonable request.
